# Strategies for prevention and control of Chagas disease: a rapid review of systematic reviews, 2024

**DOI:** 10.1590/S2237-96222026v35e20250495.en

**Published:** 2026-04-01

**Authors:** Vagner José Mendonça, Roberta Crevelário de Melo, Fábio Solon Tajra, Raimundo Leoberto Torres de Sousa, Bruno Guedes Alcoforado Aguiar

**Affiliations:** 1 Universidade Federal do Piauí, Departamento de Parasitologia e Microbiologia, Teresina, PI, Brazil Universidade Federal do Piauí Departamento de Parasitologia e Microbiologia Teresina PI Brazil; 2 Núcleo de Evidências, Instituto de Saúde da Secretaria da Saúde do Estado de São Paulo, SP, Brazil Núcleo de Evidências Instituto de Saúde da Secretaria da Saúde do Estado de São Paulo SP Brazil; 3 Universidade Federal do Piauí, Departamento de Medicina Comunitária, Teresina, PI, Brazil Universidade Federal do Piauí Departamento de Medicina Comunitária Teresina PI Brazil; 4 Fiocruz Piauí, Teresina, PI, Brazil Fiocruz Piauí Teresina PI Brazil

**Keywords:** Chagas Disease, Vector Control of Diseases, Community Participation, Public Health, Review, Enfermedad de Chagas, Control de Vectores de las Enfermedades, Participación de la Comunidad, Salud Pública, Revisión

## Abstract

**Objective:**

To identify effective strategies for the prevention and control of Chagas disease.

**Methods:**

This is a rapid review of systematic reviews. The search was conducted in March 2024, with no time restrictions, in the Medline (via PubMed) and Embase databases. Study selection was performed by pairs of reviewers using Rayyan, and data extraction was conducted in a standardized spreadsheet. Methodological quality was assessed using the Assessment of Multiple Systematic Reviews tool, version 2. Data were analyzed and presented narratively.

**Results:**

Of the 21 reviews identified, four systematic reviews were included. The first, which included 93 studies, indicated that insecticides were an effective intervention for reducing infestations. However, reinfestations were frequent, and highlighted community-based vector notification as the most cost-effective strategy compared with active surveillance or passive devices. The second addressed the monitoring of domestic and wild animals as sentinels to identify risk areas and support public policies. The third reinforced household protection with insecticides, but reported inconsistent results for insecticide-impregnated curtains and bed nets. The fourth, with only one study on Chagas disease, addressed educational actions and environmental management aimed at behavioral change and reduction of infestation. All reviews presented a critically low methodological quality.

**Conclusion:**

Control of Chagas disease remains primarily reliant on insecticides, underscoring the need for community participation as a strategic complement to ensure sustainability. Given the limited number and low methodological quality of the studies, caution is recommended when applying these findings.

## Resumen

**Objetivo:**

Identificar estrategias eficaces para la prevención y el control de la enfermedad de Chagas.

**Métodos:**

Se trata de una re­visión rápida de revisiones sistemáticas. La búsqueda se realizó sin restricción temporal en las bases de datos Medline (vía PubMed) y Embase en marzo de 2024. La selección fue realizada por pares de revisores en Rayyan, y la extracción se llevó a cabo en una hoja de cálculo estandarizada. La evaluación de la calidad metodológica se realizó mediante la herramienta Assessment of Multiple System­atic Reviews, versión 2. Los datos fueron analizados y presentados de forma narrativa.

**Resultados:**

De 21 revisiones identificadas, se incluyeron 4 revisiones sistemáticas. La primera, que reunió 93 estudios, señaló el uso de insecticidas como una intervención eficaz para reducir la infestación, aunque con reinfestaciones frecuentes, y destacó la notificación comunitaria de vectores como la estrategia con mayor costo-efectividad en comparación con la vigilancia activa o los dispositivos pasivos. La segunda abordó el monitoreo de animales domésticos y silvestres como centinelas para identificar áreas de riesgo y apoyar políticas públicas. La tercera reforzó la protección domiciliaria con insecticidas, pero mostró resultados inconsistentes con cortinas y mosquiteros impregnados. La cuarta, con solo un estudio sobre Chagas, abordó acciones educativas y de manejo ambiental orientadas al cambio conductual y a la reducción de la infestación. Todas presentaron una calidad metodológica críticamente deficiente.

**Conclusión:**

El control de la enfermedad de Chagas continúa basándose en insecticidas, lo que resalta la participación comunitaria como un complemento es­tratégico para garantizar la sostenibilidad. Debido al número limitado y a la baja calidad metodológica de los estudios, se recomienda precaución al aplicar estos hallazgos.

## Introduction

Chagas disease, also known as American trypanosomiasis, is a socially determined disease ^1^ and is caused by the protozoan *Trypanosoma cruzi*. Other determinants include the vector's habitat, socioeconomic conditions, climate and environment, public health control practices, and population mobility ^1^^,^^5^^,^^6^.

Despite being known the beginning of the 20th century Chagas disease remains a neglected disease ^7^. This means that it receives little attention and limited financial resources compared with other diseases, resulting in significant gaps in research, treatment development, and effective public health policies. 

Available treatments are limited, are effective mainly in the acute phase of the disease, and there is no preventive vaccine. People affected by the disease experience invisibility and call for sensitive listening, given the need to recognize their demands ^8^^,^^9^. Control efforts focus on environmental interventions and health education; However, they still face considerable challenges, especially in rural areas and low-income communities, with persistent reinfestations by triatomine bugs (kissing bugs) posing a major challenge ^10^.

In Latin America, an estimated 5.3 million people were infected with *Trypanosoma cruzi* in 2010 ^7^. In Brazil, the mortality burden of Chagas disease is high and represents one of the four leading causes of death from infectious and parasitic diseases. Since 2015, an average of 4 thousand deaths per year have been recorded with the disease as the underlying cause ^7^^,^^10^. Despite its epidemiological and social relevance, important gaps persist in the available evidence on the prevention and control of Chagas disease, hindering the definition of more effective and sustainable approaches. 

This study sought to map existing systematic reviews in the literature and identify research gaps and priorities. The objective was to identify effective strategies for the prevention and control of Chagas disease.

## Methods

Study design

This is a rapid review of systematic reviews, a systematic method that optimizes the review process by leveraging validated methodological shortcuts to meet policymakers' needs. Rapid reviews of systematic reviews aim to meet demand in health system management, in which strategies identified in systematic reviews are used to address health problems ^11^. 

To ensure timely delivery to decision-makers, validated methodological shortcuts were adopted, considering that full reviews are difficult to implement in health management settings. These shortcuts enabled feasible delivery and provided valuable, reliable information through rapid responses ^11^. 

Among the shortcuts adopted in this review, the extraction process and the assessment of the methodological quality of the systematic reviews stood out; these were conducted by one researcher and reviewed by another. No assessment of confidence in the systematic reviews was performed.

Eligibility criteria

To guide this rapid review of systematic reviews, the following research question was formulated: "Which strategies are effective for the prevention and control of Chagas disease?" This question was structured according to the PICOS acronym (detailed below), which is widely used to formulate clinical questions and define inclusion criteria in systematic reviews.

• P (population/problem): refers to individuals exposed to Chagas disease, considering its incidence and prevalence in different endemic regions. 

• I (intervention): encompasses strategies for the prevention and control of the disease, whether applied individually or in combination, including epidemiological surveillance actions, vector control, health education, and housing improvements. 

• C (comparator): no comparator was applied, as the objective of the review was to map and describe existing interventions, regardless of comparison groups. 

• O (*outcome*): includes reduction of the population of triatomine vectors, decrease in disease incidence, increased population knowledge about the disease, and identification of possible adverse events resulting from the interventions. 

• S (*study*/study type): considers systematic reviews the methodological design of choice, given their robustness in synthesizing scientific evidence.

Systematic reviews published in English, Spanish, and Portuguese, with no temporal or geographic restrictions, that addressed strategies for the prevention and control of Chagas disease were considered eligible. Publications such as conference abstracts, primary and tertiary studies, theses and dissertations, or those addressing strategies related to other vectors were excluded.

Sources of information

Searches were conducted in the scientific literature databases Medline (via PubMed) and Embase. No eligible studies were found in other indexed databases. No filters were applied in the databases regarding year of publication, publication status, or language. Systematic review filters were applied in the databases to optimize the rapid review process.

Search strategy

A search strategy was developed for the Medline database (via PubMed) and adapted for the Embase database. The search strategies were elaborated based on the combination of keywords structured according to the PICOS acronym, using MeSH terms in Medline (via PubMed) and Emtree terms in Embase, combined with appropriate Boolean operators (Supplementary Table 1). Searches in all databases were conducted on 19 March 2024, and the results were exported to the bibliographic management application Rayyan ^12^. 

Selection process

After removing duplicate studies, four researchers (BG, FST, RLTS, and VJM) independently selected studies based on titles and abstracts and subsequently assessed the full texts using Rayyan ^12^. One reviewer (RCM) checked all selections, and discrepancies were resolved by consensus through an online meeting. 

Data collection process

A prior alignment among the researchers was carried out using a standardized electronic spreadsheet. Four researchers (BG, FST, RLTS, and VJM) independently extracted data from the included studies. One reviewer (RCM) checked all extractions. Discrepancies were resolved by consensus through an online meeting. 

Data items

Data were extracted on: authors; year of publication; countries; study settings; study type; name of the intervention; frequency of implementation; target population of the intervention; intervention setting; and observed outcomes.

Methodological assessment

The quality of the included systematic reviews was assessed by four independent reviewers (BG, FST, RLTS, and VJM) using the Assessment of Multiple Systematic Reviews tool, version 2 (AMSTAR 2) ^13^. Discrepancies were discussed and resolved with one author (RCM). The final classification was performed according to the presence of flaws in critical and non-critical items described by the authors ^13^, as follows: 

• High confidence: no critical flaws and at most one non-critical flaw. 

• Moderate confidence: no critical flaws and more than one non-critical flaw. 

• Low confidence: one critical flaw, with or without non-critical flaws.

• Critically low confidence: more than one critical flaw, with or without non-critical flaws.

Data synthesis methods

Data synthesis was conducted narratively, grouping findings by type of intervention identified in the included reviews. Reported effects, consistency of evidence, and application contexts were described, highlighting methodological gaps and limitations. The analysis aimed to identify patterns of effectiveness, promising strategies, and contextual factors influencing the results.

## Outcomes

The search for systematic reviews on the prevention and control of Chagas disease yielded 21 publications. After removing duplicates, 19 titles and abstracts were assessed, and seven eligible studies were read in full; 4 systematic reviews were included (Figure 1). The list of excluded studies is provided in the supplement (Supplementary Table 2). 

The included systematic reviews ^14^^-^^17^ had participant samples in the primary studies ranging from 37.0 thousand households ^16^ to 32.7 thousand households ^14^ (Table 1). The strategies to address the problem consisted of insecticide spraying ^14^^,^^16^ or the use of curtains and bed nets ^16^^,^^17^; surveillance strategies with resident notification or using devices for passive vector detection ^14^; surveillance through domestic and wild animals as sentinels ^17^; and intersectoral collaboration strategies that included health education ^15^. Four primary studies were included in more than one systematic review (Table 2). 

The included systematic reviews were classified as critically low methodological quality according to the AMSTAR 2 (Figure 2). Recurring flaws were observed in the adherence to critical domains, particularly the following aspects: lack of explicit reporting on the prior definition of review methods and justification for significant protocol deviations (item 2); lack of consideration of the risk of bias of included studies in the interpretation and discussion of results (item 13); failure to present the list of excluded studies with justification for each exclusion (item 7); and inadequate or absent techniques for assessing the risk of bias of included studies (item 9).

The primary studies included in the systematic reviews were conducted predominantly in Brazil (34.7%) and Argentina (22.2%). Other countries were less represented: Guatemala (8.3%); Chile and Venezuela (both 6.9%); the United States (4.2%); Bolivia, Mexico, Nicaragua, and Paraguay (each 2.8%); and Colombia, Honduras, Peru, and Uruguay (1.4% each).

Two systematic reviews ^14^^,^^17^ reported results regarding surveillance strategies. One concerned vector notification by residents, and the other concerned the use of sentinel animals.

One of the systematic reviews analyzed results from 93 articles on interventions for Chagas disease control and surveillance ^14^. Six studies conducted in Brazil, Argentina, Chile, and Venezuela were used to compare resident-based vector notification with active vector searches performed by professional health surveillance teams ^14^. The data indicated that resident-based vector notification outperformed active insect searches conducted by surveillance teams in detecting infestation foci. Surveillance systems were more effective when residents reported suspected insects to the surveillance team than when the program team conducted searches, whether manually or with vector-detection devices. 

**Figure 1:**
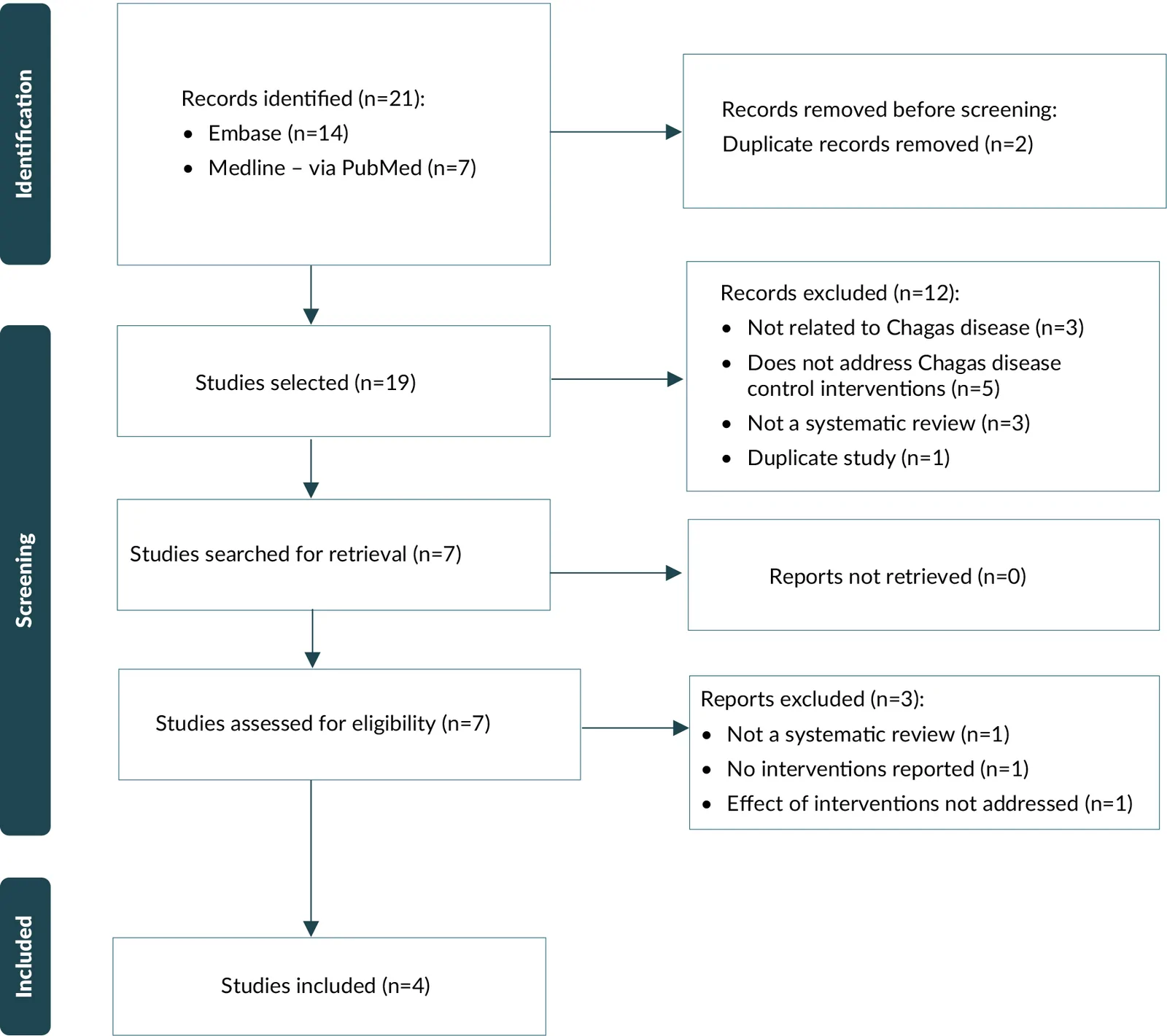
Selection and inclusion of studies in the review

**Table 1:** Characteristics of included studies (n=4)

Review (author, year)	Study design	Number of studies on Chagas disease	Countries	Number of households	Intervention	Outcomes
Abad-Franch, 2011	Systematic review	93	Brazil, Argentina, Paraguay, Uruguay, Chile, Bolivia, Venezuela, Nicaragua, Honduras, Guatemala, Mexico, and Peru	100 to 32 thousand households	Insecticide spraying in households Community-based vector notification	Indoor spraying with pyrethroids rapidly reduced infestation by ~90.0%-100.0% in the first year (from 60.0%-80.0% to <5.0%), while peridomiciliary reduction was more modest (~50.0%-70.0%) and less durable. Resident notification was the most effective form of Chagas disease surveillance, detecting 4 to 7 times more vectors than active searches by community health agents and, in some scenarios, over 100 times more than passive detection devices.
Herdiana, 2018	Systematic review	1	Guatemala	248 households	Intersectoral collaboration (insecticide application, community education on Chagas disease and risk factors, and participatory rodent control with organic waste management).	Community knowledge about Chagas disease, its transmission, and prevention methods (no aggregated numerical data). Preventive behaviors adopted by families in intervention areas, associated with environmental and peridomiciliary management (no aggregated numerical data).
Horstick & Runge-Ranzinger, 2018	Systematic review	32	Argentina, Brazil, Guatemala, Venezuela, and Colombia	37 to 1,541 households	Insecticide spraying Curtains treated with lambda-cyhalothrin Treated bed nets	Household infestation reduced from 38.0% to 0.8% (cypermethrin) and from 37.0% to 0.5% (deltamethrin) after 13 months. Household infestation reduced from 20.7% before the intervention to 6.8% after 12 months. Curtains treated with lambda-cyhalothrin (12.5 mg/m²) eliminated vectors in rooms but did not reduce infestation at the household level compared with control (no aggregated numerical data). Presence of triatomines: 75.0% in the intervention versus 86.4% in the control (Venezuela) and 12.0% in the intervention vs. 27.3% in the control (Colombia).
Kruse, 2019	Systematic review	41	United States	Data aggregated by region (no household numbers provided).	Use of domestic and wild animals as sentinels	Populations of animals infected with Trypanosoma cruzi allowed the identification of households and groups susceptible to Chagas disease transmission (no aggregated numerical data). Seropositive animals were able to predict the resurgence of Trypanosoma cruzi populations, as they were spatially linked to Chagas infection zones and areas with vector presence (no aggregated numerical data).

**Table 2:** Overlap matrix among assessed systematic reviews (n=4)

	Systematic reviews included			
Primary studies used in systematic reviews focusing on Chagas disease	Abad, 2011	Herdiana, 2018	Horstick, 2018	Kruse, 2019
Acevedo, 2000; Aché, 2001; Ávila, 1999; Campbell, 2007; Candioti, 1997; Cardinal, 2006; Cardinal, 2007; Carneiro, 1994; Carneiro, 2002; Cécere, 1997; Cécere, 1999; Cécere, 2002; Cécere, 2004; Cécere, 2006; Cedillos, 1998; Chuit, 1992; Cohen, 2006; Cuba, 2003; Dias, 1968; Dias, 2005; Diotaiuti, 1994; Diotaiuti, 2000; Dumonteil, 2004; Dumonteil, 2009; Falavigna, 2002; Feliciangeli, 2007; Ferro, 1995; Fleming, 1992; Forattini, 1969; Forattini, 1973; Forattini, 1983; Forattini, 1983; García, 1988; García, 1992; García, 1993; Gómez, 1965; González, 1996; Guillén, 1997; Gürtler, 1993; Gürtler, 1994; Gürtler, 1995; Gürtler, 1999; Gürtler, 2001; Gürtler, 2007; Hashimoto, 2006; Jörg, 1989; Lima, 1990; Lorca, 1996; Lorca, 2001; Maluf, 1970; Marco, 1999; Marcondes, 1989; Monroy, 2009; Nakagawa, 2003; Nakagawa, 2003; Neghme, 1991; Oliveira, 1996; Oliveira, 2000; Oliveira Filho, 2000; Palma, 1996; Passos, 1997; Paulone, 1988; Piesman, 1984; Sosa, 2009; Tonn, 1976; Vázquez, 2002; Vázquez, 2005; Villela, 2005; Villela, 2009; Wanderley, 1991; Wastavino, 2004; Wisnivesky, 1987; Wisnivesky, 1993; Zeledón, 2006; Zeledón, 2008; Zerba, 1997	x			
Agapova, 2010; Castillo, 2008; Castro, 2009; Hanford, 2007; Lee, 2013; Manne, 2016; Perez, 2015; Piron, 2008; Roque, 2008; Rosypal, 2011; Sánchez, 2016; Schmunis, 2010; Soriano, 2014; Soriano, 2016; Woodhall, 2014				x
Cecere, 2013; Oliveira, 2000			x	
De Urioste-Stone, 2015		x	x	
Gürtler, 2004; Herber, 2003; Kroeger, 2003	x		x	
Tenney, 2014	x			x

**Figure 2:**
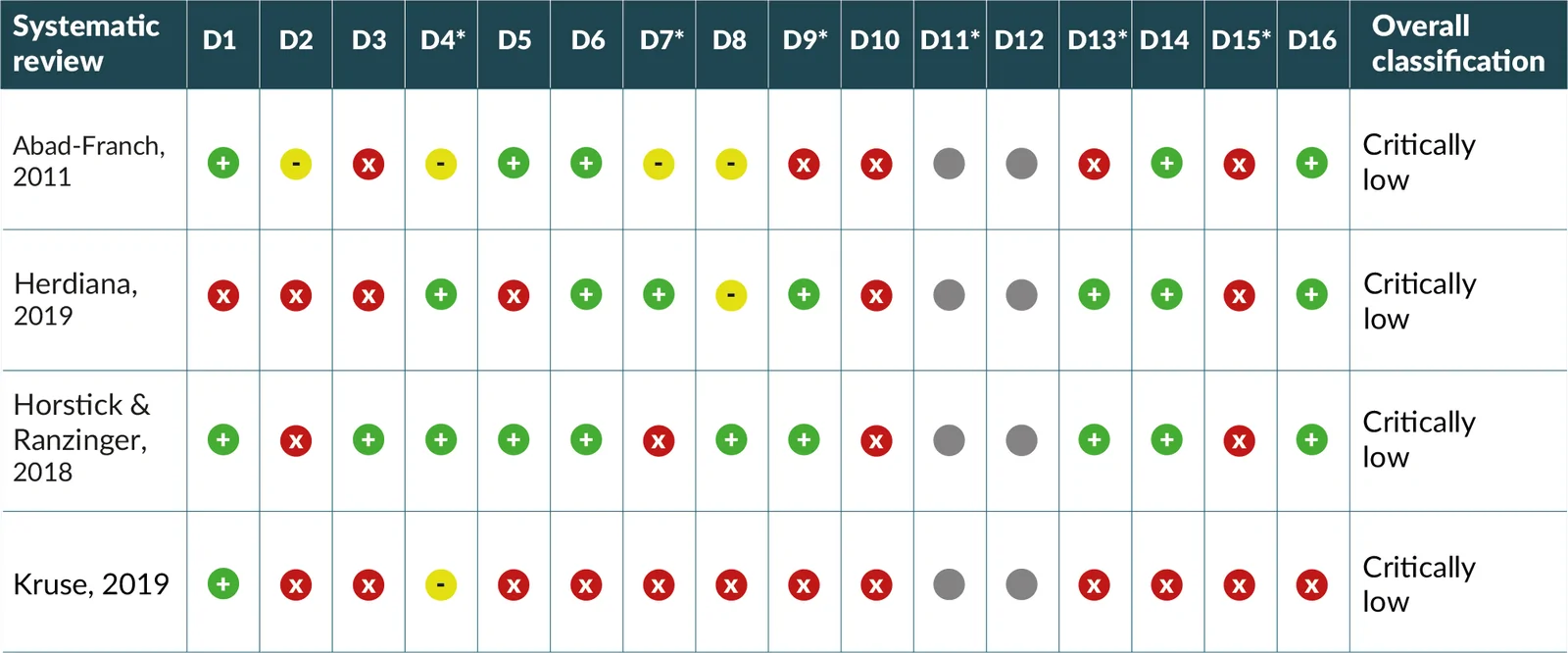
Assessment of the quality of included studies (n=4) Note: * indicates critical domains for classification; ✓: yes; ✗: no; ~: partially yes; NA: meta-analysis not performed.

This review highlighted that resident participation in vector notification should be a strategic component of Chagas disease surveillance. Still, only professional insecticide spraying has consistently been effective in eliminating infestation foci ^14^. Since residents are asked to report suspected insect infestations in their homes, there is a need for rapid response by surveillance professionals to eliminate infestations when necessary ^14^.

Vector detection devices, such as Gómez-Núñez boxes, María sensor boxes, and other devices to capture triatomines (kissing bugs) or detect their traces, were also mentioned. Although sometimes complementary to active searches by surveillance teams, they proved inferior to resident-based notification ^14^. 

The use of dogs, as well as other domestic and wild animals, as sentinels was reported in one of the included reviews ^17^. These animals can be used to track risk zones and areas with potential transmission or occurrence of Chagas disease ^17^. Sentinel animals are employed to detect disease circulation and provide early warning of outbreaks, allowing the implementation of preventive measures. This can be more cost-effective than other monitoring methods, such as the capture and analysis of triatomines. The main justifications for using animals for this purpose are their resistance to infection and ease of handling. However, such use requires ethical care and adherence to management and monitoring protocols to minimize animal suffering. Monitoring is performed using blood or other bodily fluids from animals, which are analyzed for antibodies against *Trypanosoma cruzi* or for the parasite itself.

Sentinel animals infected with *Trypanosoma cruzi* enabled the identification of households and groups at risk of Chagas disease transmission. These sentinels predicted the resurgence of *Trypanosoma cruzi* populations, as they were spatially linked to zones of Chagas disease infection and to areas with vector presence ^17^.

Two systematic reviews ^14^^,^^16^ reported results on the use of insecticides and their effects on infection rates among vectors and hosts.

Analysis of more than 90 studies conducted in several countries, particularly in Latin America, revealed important patterns across triatomine species (kissing bugs) ^14^. The results showed unequivocally that domestic insecticide spraying successfully reduced infestation rates throughout Latin America. The review, however, warned that reinfestation of dwellings by native vector species is common. In many cases, the elimination of introduced populations was closely followed by the occupation of vacant niches by "secondary" vector species. The review presented studies addressing both vector reduction and significant decreases in seropositivity rates in countries that adopted this strategy ^14^.

The most widely used and successfully applied intervention for Chagas disease was insecticide spraying, both intra- and peridomiciliary ^16^. This intervention successfully reduced Chagas disease transmission in the Southern Cone. Although the systematic review supported this intervention, reliance on a single intervention for protection against Chagas disease is common, often using the same insecticides. This creates an opportunity for the development of resistance and the formation of new vector habitats ^16^.

One systematic review ^15^ highlighted the importance of intersectoral collaboration in reducing vector-borne diseases, including malaria, dengue, Zika, schistosomiasis, African trypanosomiasis, leishmaniasis, Chagas disease, yellow fever, Japanese encephalitis, and onchocerciasis. Only one of the included studies addressed Chagas disease. This study emphasized the importance of educational interventions and social mobilization, supported by community leaders, along with environmental management (rodent control, composting, home gardens), to promote behavioral change and reduce infestations ^15^.

## Discussion

This rapid review included four systematic reviews that addressed strategies for the prevention and control of Chagas disease. The interventions were interpreted into three options for addressing the problem: (i) surveillance strategies (entomological surveillance with resident-based notification and the use of domestic and wild animals as sentinels); (ii) chemical control strategies (insecticide spraying and insecticide-impregnated curtains); and (iii) intersectoral actions (education combined with environmental strategies).

The listed strategies may be implemented individually or in combination, according to the local context. The results of this review were derived mainly from studies conducted in Latin America, reflecting the epidemiological reality of endemic areas or areas with reemergence of transmission. However, it is important to consider that the data came from a small sample of systematic reviews and their included studies. 

Resident-based notification is a critical component in the control of Chagas disease, as it promotes "immediate" or "near-immediate" detection, strengthens vector surveillance and control, engages the community, and enables essential feedback for health policy formulation ^18^. Active collaboration by residents is fundamental to an effective and sustainable response to the disease. Residents provide valuable information on local conditions that is essential to territorialization and the identification of risk areas. 

In addition, residents can help identify and report triatomine (kissing bug) infestations and report suspected cases of the disease. This creates a network of support and collaboration between residents and health authorities, facilitating the implementation of control and prevention strategies ^19^. Such interactions are essential for adapting vector control and elimination measures in a more targeted and efficient manner and for supporting the development of health policies tailored to the specific needs of each community.

Intersectoral collaboration integrates sectors (health, education, environment, and social development) and enables the combination of knowledge and resources essential to the control of Chagas disease. Health education, for example, is fundamental for sharing knowledge and practices related to the disease and its vectors. Collective health education actions that combine information on the biology of triatomines (kissing bugs) and prevention practices can be strategic in the context of Chagas disease. This can be achieved by implementing educational programs in schools and communities, as well as through social media ^19^^,^^20^.

Other possibilities may include environmental control strategies, such as improving housing conditions and implementing vector control measures. Integration with the sanitation and urbanization sectors may result in the elimination of suitable habitats for insect vectors. In Brazil, housing improvements constitute a structural measure with significant impact, as evidenced by the elimination or reduction of infestations across different contexts. Still, they also entail operational difficulties that impede sustainability ^21^^-^^24^. 

At the national level, the sustainability of intradomiciliary insecticide spraying is ensured through centralized procurement by the Ministry of Health, which distributes products to the states and coordinates logistics with municipalities ^25^. This process ensures a certain degree of stability. Still, it also depends on administrative processes and coordination across different levels of management, which may compromise the continuity of actions in scenarios of poor coordination. 

For participatory surveillance, triatomine information points (popularly known as PIT [*postos de informação de triatomíneos* ]) are strategic for expanding community participation in Chagas disease surveillance, thereby favoring early vector detection. However, their effectiveness is limited by implementation and sustainability challenges, including low visibility, lack of records, frequent deactivation, and the absence of feedback to the population. These barriers highlight the need for greater coordination of health services and educational campaigns to ensure the active and continuous use of triatomine information points ^26^.

Although the results indicated promising interventions for the prevention and control of Chagas disease, the included systematic reviews exhibited methodological flaws and were classified as critically low. These findings point to important methodological gaps that should be considered by those conducting future systematic reviews on the topic. It was noted that systematic reviews in this area should be conducted using rigorous methods, which are essential to enhance the reliability and applicability of the evidence produced.

This study also presented limitations inherent to the design of a rapid review: although based on validated methods, it employs methodological shortcuts to provide timely responses to decision-makers in the short term. A preliminary search of several databases identified only two potentially eligible studies, which were included in the study. Methodological quality assessment was performed by one researcher and validated by another. No assessment of confidence in the systematic reviews was performed.

It should be noted that significant gaps were identified. The limited number and quality of the systematic reviews identified and analyzed highlight the need for more rigorous reviews on the prevention and control of Chagas disease. None of the analyzed systematic reviews reported actions to improve prenatal care or reduce vertical and oral transmission of the disease, underscoring the need for further systematic reviews in this area. Although studies of this nature address the vertical transmission of Chagas disease, most focus on estimating maternal prevalence, congenital transmission rates ^27^^-^^30^, or associated risk factors ^31^. 

In addition to the study's search and review period, a systematic review focused on preventing vertical transmission was identified ^32^. This review demonstrated that trypanocidal treatment in infected women of reproductive age is an effective strategy for preventing congenital Chagas disease, with complete elimination of vertical transmission ^32^. None of the systematic reviews reported interventions related to housing improvement. 

The findings of this study point to prevention and control strategies that involve community participation in vector notification, combined with rapid responses by decentralized surveillance teams to enable the professional application of insecticides. The use of insecticide-impregnated curtains and bed nets to reduce vector infestations and the use of sentinel animals to track Chagas disease incidence were also highlighted. Active surveillance was also mentioned, with or without vector-detection devices to support it. Finally, intersectoral actions involving the health sector, universities and research institutions, international donors, non-governmental organizations, communities, environmental sectors, and health educators should be considered in the formulation of prevention and control strategies for Chagas disease to reflect the problem as a health, environmental, and social issue. 

## Data Availability

The study used bibliographic and publicly accessible data. The spreadsheet containing data extraction and the Assessment of Multiple Systematic Reviews, version 2, assessment is available in a supplementary file at: https://osf.io/5vuar.
