# Supplementary material for: Strategies for prevention and control of Chagas disease: a rapid review of systematic reviews, 2024
Source: Epidemiol Serv Saude. 2026 Apr 1;35:e20250495. doi: 10.1590/S2237-96222026v35e20250495.en (PMC13686350; doi:10.1590/S2237-96222026v35e20250495.en)
Supplement: Supplementary file 2 [file 2237-9622-ress-35-e20250495-Supplementary-table2.pdf]

Supplementary Table 2. Reason for exclusion of assessed studies

Stage	Justification for exclusion	Study
Title and abstract screening	Not related to Chagas disease	Monnier N, Barth-Jaeggi T, Knopp S, Steinmann P. Core components, concepts and strategies for parasitic and vector-borne disease elimination with a focus on schistosomiasis: a landscape analysis. PLoS Negl Trop Dis. 2020;14(10):e0008837. doi:10.1371/journal.pntd.0008837.
Title and abstract screening	Not related to Chagas disease	Weill A, Bernigaud C, Mokni M, Gil S, Elefant E, Chosidow O. Scabies-infested pregnant women: a critical therapeutic challenge. PLoS Negl Trop Dis. 2021;15(1):e0008929. doi:10.1371/journal.pntd.0008929.
Title and abstract screening	Not related to Chagas disease	Wilson AL, Dhiman RC, Kitron U, Scott TW, van den Berg H, Lindsay SW. Benefit of insecticide-treated nets, curtains and screening on vector borne diseases, excluding malaria: a systematic review and meta-analysis. PLoS Negl Trop Dis. 2014;8(10):e3228. doi:10.1371/journal.pntd.0003228.
Title and abstract screening	Does not address Chagas disease control interventions	Carpio A, Romo ML, Parkhouse RME, Short B, Dua T. Parasitic diseases of the central nervous system: Lessons for clinicians and policy makers. Expert Rev Neurother. 2016;16(4):401-14. https://doi.org/10.1586/14737175.2016.1155454
Title and abstract screening	Does not address Chagas disease control interventions	Golgher D, Gazzinelli RT. Innate and acquired immunity in the pathogenesis of Chagas disease. Autoimmunity. 2004;37(5):399-409. https://doi.org/10.1080/08916930410001713115
Title and abstract screening	Does not address Chagas disease control interventions	Higuita NIA, Franco-Paredes C, Henao-Martinez AF, Beatty NL, Manne-Goehler J, Forsyth CJ. Chagas disease and domestic medical screening guidance for newly arrived individuals under a humanitarian-based immigration status: a call for action. Am J Trop Med Hyg. 2022;107(5):960-3. doi:10.4269/ajtmh.22-0309.
Title and abstract screening	Does not address Chagas disease control interventions	Marin-Neto JA, Cunha-Neto E, Marciel BC, Simões MV. Pathogenesis of chronic Chagas heart disease. Circulation. 2007;115(9):1109-23. https://doi.org/10.1161/circulationaha.106.624296
Title and abstract screening	Does not address Chagas disease control interventions	Nogueira SS, Felizardo AA, Caldas IS, Gonçalves RV, Novaes RD. Challenges of immunosuppressive and antitrypanosomal drug therapy after heart transplantation in patients with chronic Chagas disease: A systematic review of

		clinical recommendations. Transplant Rev. 2018;32(3):157. https://doi.org/10.1016/j.trre.2018.04.003
Title and abstract screening	Not a systematic review	Ackley C, Elsheikh M, Zaman S. Scoping review of neglected tropical disease interventions and health promotion: A framework for successful NTD interventions as evidenced by the literature. PLoS NTD. 2021;15(7). https://doi.org/10.1371/journal.pntd.0009278
Title and abstract screening	Not a systematic review	Bello Corassa R, Aceijas C, Alves PAB, Garelick H. Evolution of Chagas' disease in Brazil. Epidemiological perspective and challenges for the future: A critical review. Perspect Public Health. 2017;137(5):289-95. https://doi.org/10.1177/1757913916671160
Title and abstract screening	Not a systematic review	Soriano-Arandes A, Angheben A, Serre-Delcor N, Treviño-Maruri B, Prat JGI, Jackson Y. Control and management of congenital Chagas disease in Europe and other non-endemic countries: Current policies and practices. Trop Med Int Health. 2016;21(5):590-6. https://doi.org/10.1111/tmi.12687
Title and abstract screening	Duplicate study	Abad-Franch F, Vega MC, Rolón MS, Santos WS, Arias AR. Community Participation in Chagas Disease Vector Surveillance: Systematic Review. PLoS Negl Trop Dis. 2011;5(6):e1207. https://doi.org/10.1371/journal.pntd.0001207
Full-text reading	No interventions reported	Coura JR, Borges-Pereira J. Chagas disease. What is known and what should be improved: A systemic review. 2012;45(3):286-96. https://doi.org/10.1590/S0037-86822012000300002
Full-text reading	Effect of interventions not addressed	Sanmartino M, Mateyca C, Pastorino IC. What are we talking about when we talk about education and Chagas? A systematic review of the issue. Biochim Biophys Acta Mol Basis Dis. 2020;1866(5):165691. https://doi.org/10.1016/j.bbadis.2020.165691
Full-text reading	Not a systematic review	Standley C, Boyce MR, Klineberg A, Essix G, Katz R. Organization of oversight for integrated control of neglected tropical diseases within Ministries of Health. PLoS Negl Trop Dis [Internet]. 2018;12(11). Available at: https://www.embase.com/search/results?subaction=viewrecord&id=L625338509&from=export U2 - L625338509
