# Supplementary material for: Strategies for prevention and control of Chagas disease: a rapid review of systematic reviews, 2024
Source: Epidemiol Serv Saude. 2026 Apr 1;35:e20250495. doi: 10.1590/S2237-96222026v35e20250495.en (PMC13686350; doi:10.1590/S2237-96222026v35e20250495.en)
Supplement: Supplementary file 3 [file 2237-9622-ress-35-e20250495-suplementar-tabela1.pdf]

Tabela Suplementar 1. Estratégia de busca utilizada em cada base indexada

**Base**	**Estratégia**	**Data da busca**	**Nº de estudos**
Embase	('chagas disease'/exp OR 'american trypanosomiasis' OR 'chagas disease' OR 'chagas infection' OR 'chagas mazza disease' OR 'chagas`s disease' OR 'trypanosoma cruzi infection' OR 'disease, chagas' OR 'infection by trypanosoma cruzi' OR 'infection of trypanosoma cruzi' OR 'south american trypanosomiasis' OR 'trypanosomiasis, american' OR 'trypanosomiasis, south american') AND ('communicable disease control'/exp OR 'communicable disease control') AND [embase]/lim AND [medline]/lim AND 'systematic review'	19/3/2024	14
PubMed	((((((((((Chagas Disease[MeSH Terms]) OR (Chagas Disease[Title/Abstract])) OR (American Trypanosomiasis[Title/Abstract])) OR (Trypanosomiasis, American[Title/Abstract])) OR (Trypanosomiasis, South American[Title/Abstract])) OR (South American Trypanosomiasis[Title/Abstract])) OR (Trypanosoma cruzi Infection[Title/Abstract])) OR (Infection, Trypanosoma cruzi[Title/Abstract])) OR (Infections, Trypanosoma cruzi[Title/Abstract])) OR (Trypanosoma cruzi Infections[Title/Abstract])) OR (Chagas' Disease[Title/Abstract])) AND ((((((((Communicable Disease Control[MeSH Terms]) OR (Communicable Disease Control[Title/Abstract])) OR (Biosecurity[Title/Abstract])) OR (Disease Reservoirs[Title/Abstract])) OR (Disease Vectors[Title/Abstract])) OR (Infection Control[Title/Abstract])) OR (Infectious Disease Medicine[Title/Abstract])) OR (Disease Eradication[Title/Abstract])) Filters: Systematic Review	19/3/2024	7
**Total**			**21**
