# Supplementary material for: Strategies for prevention and control of Chagas disease: a rapid review of systematic reviews, 2024
Source: Epidemiol Serv Saude. 2026 Apr 1;35:e20250495. doi: 10.1590/S2237-96222026v35e20250495.en (PMC13686350; doi:10.1590/S2237-96222026v35e20250495.en)
Supplement: Supplementary file 4 [file 2237-9622-ress-35-e20250495-suplementar-tabela2.pdf]

**Tabela Suplementar 2.** Motivo da exclusão dos estudos avaliados

| **Etapa** | **Justificativa para exclusão** | **Estudo** |
|---|---|---|
| Leitura de título e resumo | Não é sobre doença de Chagas | Monnier N, Barth-Jaeggi T, Knopp S, Steinmann P. Core components, concepts and strategies for parasitic and vector-borne disease elimination with a focus on schistosomiasis: a landscape analysis. PLoS Negl Trop Dis. 2020;14(10):e0008837. doi:10.1371/journal.pntd.0008837. |
| Leitura de título e resumo | Não é sobre doença de Chagas | Weill A, Bernigaud C, Mokni M, Gil S, Elefant E, Chosidow O. Scabies-infested pregnant women: a critical therapeutic challenge. PLoS Negl Trop Dis. 2021;15(1):e0008929. doi:10.1371/journal.pntd.0008929. |
| Leitura de título e resumo | Não é sobre doença de Chagas | Wilson AL, Dhiman RC, Kitron U, Scott TW, van den Berg H, Lindsay SW. Benefit of insecticide-treated nets, curtains and screening on vector borne diseases, excluding malaria: a systematic review and meta-analysis. PLoS Negl Trop Dis. 2014;8(10):e3228. doi:10.1371/journal.pntd.0003228. |
| Leitura de título e resumo | Não aborda intervenções de controle de doença de Chagas | Carpio A, Romo ML, Parkhouse RME, Short B, Dua T. Parasitic diseases of the central nervous system: Lessons for clinicians and policy makers. Expert Rev Neurother. 2016;16(4):401-14. https://doi.org/10.1586/14737175.2016.1155454 |
| Leitura de título e resumo | Não aborda intervenções de controle de doença de Chagas | Golgher D, Gazzinelli RT. Innate and acquired immunity in the pathogenesis of Chagas disease. Autoimmunity. 2004;37(5):399-409. https://doi.org/10.1080/08916930410001713115 |
| Leitura de título e resumo | Não aborda intervenções de controle de doença de Chagas | Higuita NIA, Franco-Paredes C, Henao-Martinez AF, Beatty NL, Manne-Goehler J, Forsyth CJ. Chagas disease and domestic medical screening guidance for newly arrived individuals under a humanitarian-based immigration status: a call for action. Am J Trop Med Hyg. 2022;107(5):960-3. doi:10.4269/ajtmh.22-0309. |
| Leitura de título e resumo | Não aborda intervenções de controle de doença de Chagas | Marin-Neto JA, Cunha-Neto E, Marciel BC, Simões MV. Pathogenesis of chronic Chagas heart disease. Circulation. 2007;115(9):1109-23. https://doi.org/10.1161/circulationaha.106.624296 |
| Leitura de título e resumo | Não aborda intervenções de controle de doença de Chagas | Nogueira SS, Felizardo AA, Caldas IS, Gonçalves RV, Novaes RD. Challenges of immunosuppressive and antitrypanosomal drug therapy after heart transplantation in patients with chronic Chagas disease: A systematic review of |

| | | |
|---|---|---|
| | | clinical recommendations. Transplant Rev. 2018;32(3):157. https://doi.org/10.1016/j.trre.2018.04.003 |
| Leitura de título e resumo | Não é revisão sistemática | Ackley C, Elsheikh M, Zaman S. Scoping review of neglected tropical disease interventions and health promotion: A framework for successful NTD interventions as evidenced by the literature. PLoS NTD. 2021;15(7). https://doi.org/10.1371/journal.pntd.0009278 |
| Leitura de título e resumo | Não é revisão sistemática | Bello Corassa R, Aceijas C, Alves PAB, Garelick H. Evolution of Chagas' disease in Brazil. Epidemiological perspective and challenges for the future: A critical review. Perspect Public Health. 2017;137(5):289-95. https://doi.org/10.1177/1757913916671160 |
| Leitura de título e resumo | Não é revisão sistemática | Soriano-Arandes A, Angheben A, Serre-Delcor N, Treviño-Maruri B, Prat JGI, Jackson Y. Control and management of congenital Chagas disease in Europe and other non-endemic countries: Current policies and practices. Trop Med Int Health. 2016;21(5):590-6. https://doi.org/10.1111/tmi.12687 |
| Leitura de título e resumo | O estudo é repetido | Abad-Franch F, Vega MC, Rolón MS, Santos WS, Arias AR. Community Participation in Chagas Disease Vector Surveillance: Systematic Review. PLoS Negl Trop Dis. 2011;5(6):e1207. https://doi.org/10.1371/journal.pntd.0001207 |
| Leitura de texto completo | Não apresenta intervenções | Coura JR, Borges-Pereira J. Chagas disease. What is known and what should be improved: A systemic review. 2012;45(3):286-96. https://doi.org/10.1590/S0037-86822012000300002 |
| Leitura de texto completo | Não aborda o efeito das intervenções | Sanmartino M, Mateyca C, Pastorino IC. What are we talking about when we talk about education and Chagas? A systematic review of the issue. Biochim Biophys Acta Mol Basis Dis. 2020;1866(5):165691. https://doi.org/10.1016/j.bbadis.2020.165691 |
| Leitura de texto completo | Não é revisão sistemática | Standley C, Boyce MR, Klineberg A, Essix G, Katz R. Organization of oversight for integrated control of neglected tropical diseases within Ministries of Health. PLoS Negl Trop Dis [Internet]. 2018;12(11). Disponível em: https://www.embase.com/search/results?subaction=viewrecord&id=L625338509&from=export U2 - L625338509 |
