# Supplementary material for: Strategies for prevention and control of Chagas disease: a rapid review of systematic reviews, 2024
Source: Epidemiol Serv Saude. 2026 Apr 1;35:e20250495. doi: 10.1590/S2237-96222026v35e20250495.en (PMC13686350; doi:10.1590/S2237-96222026v35e20250495.en)
Supplement: Translation

# Translation: Estratégias para prevenção e controle da doença de Chagas: revisão rápida de revisões sistemáticas, 2024

**DOI:** 10.1590/S2237-96222026v35e20250495.pt

**Authors:** Vagner José Mendonça, Roberta Crevelário de Melo, Fábio Solon Tajra, Raimundo Leoberto Torres de Sousa, Bruno Guedes Alcoforado Aguiar

**Affiliations:** 1 Universidade Federal do Piauí, Departamento de Parasitologia e Microbiologia, Teresina, PI, Brasil Universidade Federal do Piauí Departamento de Parasitologia e Microbiologia Teresina PI Brasil; 2 Núcleo de Evidências, Instituto de Saúde da Secretaria da Saúde do Estado de São Paulo, SP, Brasil Núcleo de Evidências Instituto de Saúde da Secretaria da Saúde do Estado de São Paulo SP Brasil; 3 Universidade Federal do Piauí, Departamento de Medicina Comunitária, Teresina, PI, Brasil Universidade Federal do Piauí Departamento de Medicina Comunitária Teresina PI Brasil; 4 Fiocruz Piauí, Teresina, PI, Brasil

**Keywords:** Doença de Chagas, Controle de Vetores de Doenças, Participação da Comunidade, Saúde Pública, Revisão

## Abstract

**Objetivo:**

Identificar estratégias eficazes de prevenção e controle da doença de Chagas.

**Métodos:**

Trata-se de revisão rápida de revisões sistemáticas. A busca foi realizada, sem restrição temporal, nas bases Medline (via PubMed) e Embase em março de 2024. A seleção foi realizada por duplas de revisores no Rayyan, com extração em planilha padronizada. A avaliação da qualidade metodológica foi feita por meio da ferramenta Assessment of Multiple Systematic Reviews, versão 2. Os dados foram analisados e apresentados de forma narrativa.

**Resultados:**

De 21 revisões identificadas, 4 revisões sistemáticas foram incluídas. A primeira, que reuniu 93 estudos, apontou o uso de inseticidas como intervenção eficaz na redução da infestação, embora com reinfestações frequentes, e destacou a notificação comunitária de vetores como estratégia mais custo-efetiva em relação à vigilância ativa ou a dispositivos passivos. A segunda abordou o monitoramento de animais domésticos e silvestres como sentinelas para identificação de áreas de risco e apoio a políticas públicas. A terceira reforçou a proteção domiciliar com inseticidas, mas apresentou resultados inconsistentes para cortinas e mosquiteiros impregnados. A quarta, com apenas um estudo sobre doença de Chagas, tratou de ações educativas e manejo ambiental voltados à mudança comportamental e à redução da infestação. Todas apresentaram qualidade metodológica criticamente baixa.

**Conclusão:**

O controle da doença de Chagas continua sendo baseado em inseticidas, o que ressalta a participação comunitária como complemento estratégico para garantir a sustentabilidade. Devido ao número limitado e à baixa qualidade metodológica dos estudos, recomenda-se cautela ao aplicar esses achados.

## Introdução 

A doença de Chagas, também conhecida como tripanossomíase americana, é uma doença determinada socialmente ^1^ e tem o protozoário *Trypanosoma cruzi* como agente etiológico ^2^^-^^4^. Outros determinantes estão relacionados com o habitat do vetor, as condições socioeconômicas, o clima e o ambiente, as práticas de controle em saúde pública e a movimentação de pessoas ^1^^,^^5^^,^^6^.

Apesar de ser conhecida desde o início do século XX, a doença de Chagas continua sendo uma doença negligenciada ^7^. Isso significa que recebe pouca atenção e poucos recursos financeiros em comparação com outras doenças, o que resulta em lacunas significativas na pesquisa, no desenvolvimento de tratamentos e nas políticas de saúde pública eficazes. 

O tratamento disponível é limitado, sendo eficaz principalmente na fase aguda da doença, e não há vacina preventiva. As pessoas acometidas pela doença enfrentam a invisibilidade e demandam por escuta sensível, tendo em vista a necessidade de reconhecimento de suas demandas ^8^^,^^9^. Os esforços de controle focam intervenções ambientais e educação em saúde, mas ainda enfrentam desafios consideráveis, especialmente em áreas rurais e comunidades de baixa renda, tendo reinfestações persistentes de triatomíneos (barbeiros) como um de seus grandes desafios ^10^.

Na América Latina, estimou-se que, em 2010, mais de 5 milhões de pessoas estariam infectadas por *Trypanosoma cruzi*^7^. No Brasil, a carga de mortalidade por doença de Chagas é elevada e representa uma das quatro principais causas de mortes por doenças infecciosas e parasitárias. Desde 2015, são registrados, em média, 4 mil óbitos a cada ano, com a doença como causa básica ^7^^,^^10^. Apesar da relevância epidemiológica e social, persistem lacunas importantes nas evidências disponíveis sobre prevenção e controle da doença de Chagas, o que dificulta a definição de abordagens mais eficazes e sustentáveis. 

Este estudo buscou mapear as revisões sistemáticas existentes na literatura e identificar as lacunas e prioridades de pesquisa. Teve-se como objetivo identificar estratégias eficazes para a prevenção e o controle da doença de Chagas.

## Métodos 

Delineamento

Trata-se de revisão rápida de revisões sistemáticas, método sistemático, que otimiza o processo de revisão sistemática, com base em alguns atalhos metodológicos validados para responder às necessidades dos formuladores de políticas públicas. A revisão rápida de revisões sistemáticas têm a finalidade de atender a uma demanda da gestão do sistema de saúde no qual são identificadas estratégias a partir de revisões sistemáticas para o enfrentamento de um problema de saúde ^11^. 

Para garantir a entrega ao tomador de decisão em tempo oportuno, foram adotados atalhos metodológicos validados, considerando que revisões completas são de difícil implementação em ambientes de gestão em saúde. Esses atalhos possibilitaram uma entrega viável e forneceram informações valiosas e seguras por meio de respostas rápidas ^11^. 

Entre os atalhos adotados nesta revisão, destacaram-se o processo de extração e a avaliação da qualidade metodológica das revisões sistemáticas, os quais foram conduzidos por um pesquisador e revisados por outro. Não foi feita a avaliação da confiança das revisões sistemáticas.

Critérios de elegibilidade

Para nortear esta revisão rápida de revisões sistemáticas, foi elaborada a seguinte pergunta de pesquisa: "Quais estratégias são eficazes para a prevenção e o controle da doença de Chagas?". Essa questão foi estruturada com base no acrônimo PICOS - detalhado a seguir -, amplamente utilizado na formulação de perguntas clínicas e na definição de critérios de inclusão em revisões sistemáticas.

• P (população/problema): refere-se a indivíduos expostos à doença de Chagas, considerando sua incidência e prevalência em diferentes regiões endêmicas. 

• I (intervenção): engloba as estratégias de prevenção e controle da doença, sejam elas aplicadas de forma isolada, sejam elas aplicadas de forma combinada, incluindo ações de vigilância epidemiológica, controle vetorial, educação em saúde e melhorias habitacionais. 

• C (comparador): não se aplicou comparador, uma vez que o objetivo da revisão foi mapear e descrever as intervenções existentes, independentemente de grupos de comparação. 

• O (*outcome*/desfecho): inclui a redução da população de triatomíneos vetores, a diminuição da incidência de casos da doença, o aumento do conhecimento da população sobre a enfermidade e a identificação de possíveis eventos adversos decorrentes das intervenções. 

• S (*study*/tipo de estudo): considera revisões sistemáticas como delineamento metodológico, por sua robustez na síntese de evidências científicas.

Foram consideradas elegíveis revisões sistemáticas publicadas em inglês, espanhol e português, sem limitação temporal ou de localidade, que abordassem estratégias para a prevenção e o controle da doença de Chagas. Excluíram-se publicações como resumos de congressos, estudos primários e terciários, teses e dissertações ou aquelas que abordassem estratégias relacionadas a outros vetores.

Fontes de informação

As buscas foram realizadas nas bases de literatura científica: Medline (via PubMed) e Embase. Não foram encontrados estudos elegíveis em outras bases indexadas. Não foram aplicados filtros nas bases de dados quanto a restrições de ano ou status de publicação ou quanto ao idioma de publicação. Utilizaram-se filtros de revisão sistemática nas bases de dados para otimizar o processo de elaboração da revisão rápida.

Estratégia de busca

Uma estratégia foi desenvolvida para a base de dados Medline (via PubMed) e adaptada para a base Embase. As estratégias de buscas foram elaboradas com base na combinação de palavras-chave, estruturadas a partir do acrônimo PICOS, usando os termos MeSH na base Medline (via PubMed) e Emtree na Embase, combinados pelos operadores booleanos apropriados (Tabela Suplementar 1). As buscas em todas as bases de dados foram realizadas em 19 de março de 2024 e exportadas para o aplicativo para gerenciamento bibliográfico Rayyan ^13^. 

Processo de seleção

A partir da retirada de estudos repetidos, quatro pesquisadores (BG, FST, RLTS e VJM) selecionaram os estudos de forma independente, a partir do título e dos resumos e, em seguida, realizaram a avaliação do texto completo utilizando o Rayyan ^12^. Um revisor (RCM) realizou a checagem de todas as seleções, e as discrepâncias foram resolvidas em consenso por meio de reunião online. 

Processo de coleta de dados

Realizou-se um alinhamento prévio entre os pesquisadores com uma planilha eletrônica padronizada. Quatro pesquisadores (BG, FST, RLTS e VJM) extraíram os estudos incluídos de forma independente. Um revisor (RCM) realizou a checagem de todas as extrações. As discrepâncias foram resolvidas em consenso por meio de reunião online. 

Lista de dados

Foram extraídos dados relacionados a: autores; ano de publicação; países; tipo de estudo; nome da intervenção; frequência de realização; público-alvo da intervenção; local da intervenção; e resultado encontrado.

Avaliação da metodológica

A qualidade das revisões sistemáticas incluídas foi avaliada por quatro revisores independentes (BG, FST, RLTS e VJM) utilizando a ferramenta Assessment of Multiple Systematic Reviews, versão 2 (AMSTAR 2) ^13^. As discrepâncias foram discutidas e resolvidas com um autor (RCM). A classificação final foi realizada conforme a presença de falhas em itens críticos e não críticos descritos pelos autores ^13^, conforme a seguir. 

• Alta confiança: nenhuma falha crítica e no máximo uma falha não crítica. 

• Moderada: nenhuma falha crítica e mais de uma falha não crítica. 

• Baixa: uma falha crítica, com ou sem falhas não críticas.

• Criticamente baixa: mais de uma falha crítica, com ou sem falhas não críticas.

Métodos de síntese dos dados

A síntese dos dados foi conduzida de forma narrativa, agrupando os achados por tipo de intervenção identificada nas revisões incluídas. Foram descritos os efeitos reportados, a consistência das evidências e os contextos de aplicação, com destaque para as lacunas e limitações metodológicas. A análise buscou identificar padrões de efetividade, estratégias promissoras e fatores contextuais que influenciaram os resultados.

## Resultados 

A busca por revisões sistemáticas sobre a prevenção e o controle da doença de Chagas resultou em 21 publicações identificadas nas bases de dados. Após a exclusão de duplicatas, 19 títulos e resumos foram avaliados, e 7 estudos elegíveis foram lidos na íntegra; 4 revisões sistemáticas foram incluídas (Figura 1). Disponibilizou-se a lista de estudos excluídos no suplemento (Tabela Suplementar 2). 

As revisões sistemáticas ^14^^-^^17^ incluídas apresentaram como características uma amostra de participantes composta nos estudos primários que variou de 37,0 ^20^ a 32,7 mil domicílios ^14^ (Tabela 1). As estratégias de enfrentamento do problema foram compostas por pulverização de inseticida ^14^^,^^16^ ou por uso de cortinas e redes mosquiteiras ^16^^,^^17^; estratégias de vigilância com notificação por residentes ou com utilização de dispositivos para detecção passiva de vetores ^14^; vigilância por meio de animais domésticos e silvestres como sentinelas ^17^; e estratégias de colaboração intersetorial que incluíram educação em saúde ^15^. Foram encontrados 4 estudos primários incluídos em mais de 1 revisão sistemática (Tabela 2). 

As revisões sistemáticas incluídas apresentaram qualidade metodológica classificada como criticamente baixa, conforme o instrumento AMSTAR 2 (Figura 2). Observou-se falha recorrente no atendimento a domínios críticos, especialmente nos seguintes aspectos: ausência de relato explícitosobre a definição prévia dos métodos da revisão e justificativa para desvios significativos do protocolo (item 2); falta de consideração do risco de viés dos estudos incluídos na interpretação e discussão dos resultados (item 13); não apresentação da lista de estudos excluídos, com justificativa para cada exclusão (item 7); e uso inadequadoou ausência de técnica para avaliação do risco de viés dos estudos incluídos (item 9).

**Figura 1:** Processo de seleção e inclusão de estudos na revisão

**Tabela 1:** Características dos estudos incluídos (n=4)

Revisão (autor, ano)	Delineamento	Nº de estudos sobre a doença de Chagas	Países	Nº de domicílios	Intervenção	Resultados
Abad-Franch, 2011	Revisão sistemática	93	Brasil, Argentina, Paraguai, Uruguai, Chile, Bolívia, Venezuela, Nicarágua, Honduras, Guatemala, México e Peru	100 a 32 mil domicílios	Pulverização de inseticidas em residências Notificação de vetores com participação da comunidade	A borrifação intradomiciliar com piretroides reduz rapidamente a infestação em ~90,0%-100,0% no primeiro ano (de 60,0%-80,0% para <5,0%), enquanto no peridomicílio a queda é mais modesta (~50,0%-70,0%) e menos duradoura. Notificação por moradores foi a forma mais eficaz de vigilância da doença de Chagas, detectando de 4 a 7 vezes mais vetores do que as buscas ativas por agentes de saúde e chegando a ser mais de 100 vezes superior aos dispositivos passivos de detecção em alguns cenários.
Herdiana, 2018	Revisão sistemática	1	Guatemala	248 domicílios	Colaboração intersetorial (aplicação de inseticida, educação comunitária sobre Chagas e fatores de risco, e controle participativo de roedores com manejo de resíduos orgânicos)	Conhecimento da comunidade sobre a doença de Chagas, sua transmissão e formas de prevenção (sem dados numéricos agrupados). Comportamentos preventivos adotados pelas famílias nas áreas de intervenção, associados ao manejo ambiental e peridomiciliar (sem dados numéricos agrupados).
Horstick e Runge-Ranzinger, 2018	Revisão sistemática	32	Argentina, Brasil, Guatemala, Venezuela e Colômbia	37 a 1.541 domicílios	Pulverização de inseticidas Cortinas tratadas com lambdacialotrina Redes mosquiteiras tratadas	Infestação domiciliar reduziu de 38,0% para 0,8% (cipermetrina) e de 37,0% para 0,5% (deltametrina) após 13 meses. Infestação domiciliar de 20,7% antes da intervenção para 6,8% após 12 meses. Cortinas tratadas com lambdacialotrina (12,5 mg/m^2^) eliminaram vetores nos quartos, mas não reduziram a infestação quando avaliada em nível domiciliar comparadas ao controle (sem dados numéricos agrupados). Presença de triatomíneos de 75,0% na intervenção e 86,4% no controle (Venezuela) e 12,0% na intervenção e 27,3% no controle (Colômbia).
Kruse, 2019	Revisão sistemática	41	Estados Unidos	Dados agregados por região (sem número de domicílios)	Utilização de animais domésticos e silvestres como sentinelas	Populações de animais infectados pelo Trypanosoma cruzi permitiram a identificação de casas e grupos que eram suscetíveis à transmissão da doença de Chagas (sem dados numéricos agrupados). Animais soropositivos foram capazes de prever o ressurgimento de populações de Trypanosoma cruzi, uma vez que estavam espacialmente ligados a zonas de infecção por doença de Chagas e a áreas com presença dos vetores (sem dados numéricos agrupados).

**Tabela 2:** Matriz de sobreposição entre revisões sistemáticas avaliadas (n=4)

Estudos primários utilizados nas revisões sistemáticas com foco na doença de Chagas	Revisão sistemáticas incluídas		
	Abad, 2011	Herdiana, 2018	Horstick, 2018	Kruse, 2019
Acevedo, 2000; Aché, 2001; Ávila, 1999; Campbell, 2007; Candioti, 1997; Cardinal, 2006; Cardinal, 2007; Carneiro, 1994; Carneiro, 2002; Cécere, 1997; Cécere, 1999; Cécere, 2002; Cécere, 2004; Cécere, 2006; Cedillos, 1998; Chuit, 1992; Cohen, 2006; Cuba, 2003; Dias, 1968; Dias, 2005; Diotaiuti, 1994; Diotaiuti, 2000; Dumonteil, 2004; Dumonteil, 2009; Falavigna, 2002; Feliciangeli, 2007; Ferro, 1995; Fleming, 1992; Forattini, 1969; Forattini, 1973; Forattini, 1983; Forattini, 1983; García, 1988; García, 1992; García, 1993; Gómez, 1965; González, 1996; Guillén, 1997; Gürtler, 1993; Gürtler, 1994; Gürtler, 1995; Gürtler, 1999; Gürtler, 2001; Gürtler, 2007; Hashimoto, 2006; Jörg, 1989; Lima, 1990; Lorca, 1996; Lorca, 2001; Maluf, 1970; Marco, 1999; Marcondes, 1989; Monroy, 2009; Nakagawa, 2003; Nakagawa, 2003; Neghme, 1991; Oliveira, 1996; Oliveira, 2000; Oliveira Filho, 2000; Palma, 1996; Passos, 1997; Paulone, 1988; Piesman, 1984; Sosa, 2009; Tonn, 1976; Vázquez, 2002; Vázquez, 2005; Villela, 2005; Villela, 2009; Wanderley, 1991; Wastavino, 2004; Wisnivesky, 1987; Wisnivesky, 1993; Zeledón, 2006; Zeledón, 2008; Zerba, 1997	x			
Agapova, 2010; Castillo, 2008; Castro, 2009; Hanford, 2007; Lee, 2013; Manne, 2016; Perez, 2015; Piron, 2008; Roque, 2008; Rosypal, 2011; Sánchez, 2016; Schmunis, 2010; Soriano, 2014; Soriano, 2016; Woodhall, 2014				x
Cecere, 2013; Oliveira, 2000			x	
De Urioste-Stone, 2015		x	x	
Gürtler, 2004; Herber, 2003; Kroeger, 2003	x		x	
Tenney, 2014	x			x

**Figura 2:** Avaliação da qualidade dos estudos incluídos (n=4) **Nota:** * corresponde a domínios críticos para classificação; ✓: sim; ✗: não; ~: parcialmente sim; NA: não foi realizada metanálise.

Os estudos primários incluídos nas revisões sistemáticas foram realizados especialmente no Brasil (34,7%), seguido da Argentina (22,2%). Os demais países foram menos representativos: Guatemala (8,3%); Chile e Venezuela (ambos com 6,9%); Estados Unidos (4,2%); Bolívia, México, Nicarágua e Paraguai (cada um representando 2,8%) e Colômbia, Honduras, Peru e Uruguai (1,4% para cada país).

Duas revisões sistemáticas ^14^^,^^17^ apresentaram resultados quanto às estratégias de vigilância. Uma foi relacionada à notificação de vetores pelos residentes e outra relacionada ao uso de animais sentinelas.

Uma das revisões sistemáticas analisou resultados de 93 artigos sobre intervenções para controle e vigilância de doenças de Chagas ^14^. Seis estudos realizados no Brasil, na Argentina, no Chile e na Venezuela foram utilizados para a comparação entre a notificação de vetores feita por residentes e a busca ativa de vetores feita por equipe profissional de vigilância em saúde ^14^. Os dados apontaram que a notificação de vetores feita por residentes apresentou desempenho melhor do que as buscas ativas de insetos realizadas por equipes de vigilância na detecção de focos de infestação. Os sistemas de vigilância foram mais eficazes quando os moradores notificaram insetos suspeitos para a equipe de vigilância do que quando a equipe do programa realizou a busca - ativa manualmente ou por meio de dispositivos de detecção de vetores. 

Essa revisão assinalou que a participação dos residentes na notificação de vetores deve se tornar um componente estratégico da vigilância da doença de Chagas, mas apenas a pulverização profissional de inseticidas parece consistentemente eficaz na eliminação de focos de infestação ^14^. Uma vez que os residentes são solicitados a relatar insetos suspeitos encontrados em suas casas, destaca-se a necessidade de resposta rápida providenciada por profissionais de vigilância para eliminar a infestação, quando necessário ^14^.

Dispositivos de detecção de vetores, como Gómez-Núñez boxes, María sensor boxes e outros dispositivos para capturar triatomíneos (barbeiros) ou detectar seus rastros, também foram citados. Apesar de serem por vezes complementares na busca ativa feita pelas equipes de vigilância, mostraram-se inferiores à notificação realizada por meio de participação dos residentes na notificação ^14^. 

O uso de cães, assim como de outros animais domésticos e silvestres, como sentinelas foi reportado em uma das revisões incluídas ^17^. Esses animais podem ser utilizados para rastrear zonas de risco e áreas com potencial de transmissão ou ocorrência da doença de Chagas ^17^. Os animais sentinelas são utilizados para detectar a circulação de doenças e fornecer sinais precoces de surtos, permitindo a implementação de medidas preventivas. Pode ser uma abordagem mais econômica em comparação a outros métodos de monitoramento, como a captura e análise de barbeiros. As principais justificativas para o uso de animais para essa finalidade são a resistência destes a infecções e a facilidade de manejo. Contudo, esse uso exige cuidados éticos e atenção aos protocolos de manejo e monitoramento, para minimizar o sofrimento dos animais. O monitoramento se dá por meio de amostras de sangue ou outros fluidos corporais dos animais que são analisados para a presença de anticorpos contra *Trypanosoma cruzi* ou para o próprio parasito ^17^.

Os animais sentinelas infectados pelo *Trypanosoma cruzi* permitiram a identificação de casas e grupos que eram suscetíveis à transmissão da doença de Chagas. Essas sentinelas foram capazes de prever o ressurgimento de populações de *Trypanosoma cruzi*, uma vez que estavam espacialmente ligados a zonas de infecção por doença de Chagas e a áreas com presença dos vetores ^17^.

Duas revisões sistemáticas ^14^^,^^16^ apresentaram resultados quanto ao uso de inseticidas relacionados à redução de taxas de infecção de vetores e hospedeiros.

A análise de mais de 90 estudos realizada em diversos países, especialmente nos da América Latina, revelou padrões importantes relacionados a diferentes espécies de triatomíneos (barbeiros) ^14^. Os resultados apontaram que, inequivocamente, a pulverização doméstica de inseticidas reduziu com sucesso as taxas de infestação em toda a América Latina. A revisão advertiu, no entanto, que a reinfestação de habitações por espécies vetores nativas é comum. Em muitos casos, a eliminação das populações introduzidas foi seguida de perto pela ocupação de nichos vagos por espécies de vetores "secundárias". A revisão apresentou estudos tanto para a redução de vetores quanto para a significativa redução da taxa de soropositividade em países que utilizaram essa estratégia ^14^.

A intervenção mais utilizada e aplicada com sucesso para a doença de Chagas foi a pulverização de inseticidas, tanto intradomiciliar quanto peridomiciliar ^16^. Essa intervenção reduziu com sucesso a transmissão da doença de Chagas no Cone Sul da América Latina. Apesar de a revisão sistemática apoiar essa intervenção, foi relatado que, frequentemente, se confia em uma única intervenção para a proteção contra a doença de Chagas, geralmente utilizando os mesmos inseticidas. Isso cria uma oportunidade para o desenvolvimento de resistência e novos habitats para os vetores ^16^.

Uma revisão sistemática ^15^ destacou a importância da colaboração intersetorial para reduzir doenças transmitidas por vetores, os quais abrangem diversas enfermidades como malária, dengue, zika, esquistossomose, tripanossomíase africana, leishmaniose, doença de Chagas, febre amarela, encefalite japonesa e oncocercose. Apenas um dos estudos incluídos abordou a doença de Chagas. Este ressaltou, sobretudo, a importância de ações educativas e mobilização social apoiada por líderes comunitários juntamente com manejo ambiental (controle de roedores, compostagem, hortas) para mudanças comportamentais e redução de infestação ^15^.

## Discussão 

Esta revisão rápida incluiu quatro revisões sistemáticas que abordaram estratégias de prevenção e controle de doença de Chagas. As intervenções foram interpretadas em três opções para o enfrentamento do problema: (i) estratégias de vigilância (vigilância entomológica com notificação pelos residentes e uso de animais domésticos e silvestres como sentinelas); (ii) estratégias de controle químico (pulverização de inseticida e cortinas impregnadas com inseticidas); e (iii) ações intersetoriais (educação combinada a estratégias de ambiente).

As estratégias elencadas podem ser implementadas isoladamente ou em conjunto, de acordo com o contexto local. Os resultados desta revisão derivaram principalmente de estudos realizados na América Latina, refletindo a realidade epidemiológica de áreas endêmicas ou com reemergência da transmissão. No entanto, é importante considerar que os dados foram advindos de uma pequena amostra de revisões sistemáticas e de seus estudos incluídos. 

A notificação pelos residentes é um componente crítico no controle da doença de Chagas, pois promove a detecção "imediata" ou "quase imediata", fortalece a vigilância e o controle de vetores, engaja a comunidade e possibilita o retorno essencial para a formulação de políticas de saúde ^18^. A colaboração ativa dos residentes é fundamental para uma resposta eficaz e sustentável à doença. Residentes fornecem informações valiosas sobre as condições locais que são fundamentais para o processo de territorialização e identificação de áreas de risco. 

Aliado a isso, os residentes podem ajudar a identificar e reportar a presença de barbeiros e a elencar casos de suspeição da doença. Isso cria uma rede de apoio e colaboração entre os moradores e as autoridades de saúde, o que facilita a implementação de estratégias de controle e prevenção ^19^. São interações essenciais para adaptar medidas de controle e eliminação de vetores de forma mais direcionada e eficiente e para subsidiar a elaboração de políticas de saúde às necessidades específicas de cada comunidade.

A colaboração intersetorial envolve a integração de diferentes áreas (saúde, educação, meio ambiente e desenvolvimento social) e permite a combinação de conhecimentos e recursos que são importantes para o controle da doença de Chagas. A educação em saúde, por exemplo, é fundamental para compartilhar saberes e práticas sobre a doença e seus vetores. Ações coletivas de educação em saúde que combinam informações sobre a biologia dos triatomíneos (barbeiros) e práticas de prevenção podem ser estratégicas, quando se fala da doença de Chagas. Isso pode se materializar a partir da implementação de programas educativos em escolas e comunidades e através de mídias sociais ^19^^,^^20^.

Outras possibilidades podem envolver estratégias de controle ambiental, como a melhoria das condições habitacionais e o controle de vetores. A integração com setores de saneamento e urbanização pode resultar na eliminação de habitats adequados para os insetos transmissores. No Brasil, as melhorias habitacionais configuram medida estrutural de impacto, com evidências de eliminação ou redução da infestação em diferentes contextos, mas que também contam com dificuldades operacionais que implicam desafios à sustentabilidade ^21^^-^^24^. 

Ainda no cenário nacional, a sustentabilidade da pulverização intradomiciliar com inseticidas é garantida por meio de aquisição centralizada pelo Ministério da Saúde, que os repassa aos estados, responsáveis por articular a logística de distribuição junto aos municípios ^25^. Esse processo assegura certa estabilidade, mas também é dependente de fluxos administrativos e da coordenação entre diferentes níveis de gestão, o que pode comprometer a continuidade das ações em cenários de desarticulação. 

Para a vigilância participativa, os postos de informação de triatomíneos (popularmente conhecidos como PITs) são estratégicos para ampliar a participação comunitária na vigilância da doença de Chagas, o que favorece a detecção precoce de vetores. Contudo, sua efetividade é limitada por dificuldades de implantação e sustentabilidade, o que inclui baixa divulgação, ausência de registros, desativação frequente e falta de devolutiva à população. Esses entraves evidenciam a necessidade de maior articulação dos serviços de saúde e de campanhas educativas para garantir o uso ativo e contínuo dos postos de informação de triatomíneos ^26^.

Embora os resultados tenham indicado intervenções positivas e promissoras para a prevenção e o controle da doença de Chagas, as revisões sistemáticas incluídas apresentaram falhas metodológicas, sendo classificadas como criticamente baixa. Esses achados apontaram lacunas metodológicas importantes que devem ser consideradas por quem for conduzir revisões sistemáticas futuras sobre o tema. Notou-se a necessidade de que as revisões sistemáticas nessa temática sejam elaboradas com métodos rigorosos, fundamental para aumentar a confiabilidade e a aplicabilidade das evidências produzidas.

Este estudo apresentou limitações também inerentes ao delineamento de uma revisão rápida, que, apesar de baseada em métodos validados, utiliza atalhos metodológicos com o objetivo de fornecer respostas oportunas aos tomadores de decisão em curto prazo. Foi realizada uma busca preliminar em diversas bases de dados, das quais apenas duas retornaram estudos potencialmente elegíveis e foram incluídas na pesquisa. A avaliação da qualidade metodológica foi realizada por um pesquisador e validada por outro. Não foi feita a avaliação da confiança das revisões sistemáticas.

Ressalta-se que existem lacunas significativas identificadas. A qualidade e a quantidade limitada das revisões sistemáticas encontradas e analisadas destacam a demanda por mais e melhores revisões sistemáticas sobre a prevenção e o controle da doença de Chagas. Nenhuma das revisões sistemáticas analisadas relatou ações relacionadas à melhoria do pré-natal e às diminuições da transmissão vertical e da transmissão oral da doença, o que reforça a necessidade de revisões sistemáticas nessa área. Apesar de existirem estudos dessa natureza os quais abordam a transmissão vertical da doença de Chagas, a maioria concentra-se na estimativa de prevalência materna, nas taxas de transmissão congênita ^27^^-^^30^ ou nos fatores de risco associados ^31^. 

Em complemento ao período de busca e revisão desta pesquisa, identificou-se revisão sistemática focada na prevenção de transmissão vertical ^32^. Essa revisão demonstrou que a terapia tripanocida em mulheres infectadas em idade reprodutiva é uma estratégia eficaz para a prevenção da doença de Chagas congênita, com eliminação completa da transmissão vertical ^32^. Nenhuma das revisões sistemáticas relatou intervenções relacionadas à melhoria habitacional. 

Os achados deste estudo apontaram para estratégias de prevenção e controle que envolvam a comunidade na notificação de vetores, aliada a uma resposta rápida das equipes descentralizadas de vigilância para a aplicação profissional de inseticidas. Foram elencados também o uso de cortinas e redes mosquiteiras com impregnação de inseticidas para diminuição de infestação de vetores e o uso de animais sentinelas para rastrear ocorrências de doença de Chagas. Citou-se a busca ativa, com ou sem o uso de dispositivos de detecção de vetores para auxiliar na vigilância. Por fim, ações intersetoriais envolvendo setores da saúde, universidades e instituições de pesquisa, doadores internacionais, organizações não governamentais, comunidade, ambiente, educadores de saúde, devem ser consideradas na formulação de estratégias de prevenção e controle da doença de Chagas, a fim de refletir o problema como uma questão de saúde, ambiental e social.

## Data Availability

O estudo utilizou dados bibliográficos e de acesso público. A planilha com a extração de dados e a avaliação Assessment of Multiple Systematic Reviews, versão 2, se encontra em arquivo suplementar disponível em: https://osf.io/5vuar.

## Material Suplementar

Tabela Suplementar 1

Tabela Suplementar 2
